# P094: Impact of antibiotics changes on the incidence of bloodstream infection due to extended-spectrum beta-lactamase-producing Klebsiella pneumoniae in an Algerian neonatal intensive care unit

**DOI:** 10.1186/2047-2994-2-S1-P94

**Published:** 2013-06-20

**Authors:** A Mohamed Lamine, Fetta Sadaoui, Nora Boubechou, Abdeldjallil Bezzaoucha, Chawki Ahmed Kaddache, Rachida Boukari

**Affiliations:** 1 Department of Medicine, University of Blida, Blida, Algeria

## Introduction

*Klebsiella pneumoniae* is one of the most common nosocomial bloodstream infection (BSI) pathogens in neonatal intensive care units (NICUs) of developing countries. Its ability to produce extended-spectrum beta-lactamases (ESBLs) has caused great concern worldwide. Early studies reported that high beta-lactam antibiotic consumption was an independent risk factor for acquisition of ESBL-producing *K. pneumoniae* BSI.

## Objectives

The objective of this study was to examine the impact of the reduction of beta-lactam antibiotic consumption on the incidence of ESBL-producing *K. pneumoniae* BSI in an Algerian NICU.

## Methods

A comprehensive education campaign was undertaken in the University Hospital of Blida NICU in the beginning of 2008 to reduce the beta-lactam antibiotic consumption in this unit. To measure the impact of this campaign on the incidence of ESBL-producing *K. pneumoniae* BSI, a prospective surveillance of healthcare-associated BSI was performed from 2008 to 2010 using National Nosocomial Infection Surveillance (NNIS) System criteria. Antibiotic consumption was measured by dividing the total days of beta-lactam antibiotic consumption by the total days of patients NICU stay.

## Results

From 2008 to 2010, a total of 3842 neonates who remained in the NCIU for more than 48 hours were included in the study. These patients had total patient-days of 44,424 and total beta-lactam antibiotic-days of 25,180. Beta-lactam antibiotic consumption decreased significantly from 71.4 antibiotic-days per 100 patient-days in 2008 to 41.3 antibiotic-days per 100 patient-days in 2010 (p < .01). Incidence of ESBL-producing *K. pneumoniae* BSI decreased significantly from 3.6% in 2008 to 0.2% in 2010 (p < .01), and incidence density decreased significantly from 3.2 per 1000 patient-days in 2008 to 0.2 per 1000 patient-days in 2010 (p<.01).

## Conclusion

Our findings highlight the need to minimise unnecessary and inappropriate antimicrobial use (specifically that of beta-lactam antibiotics) to prevent the acquisition of ESBL-producing *K. pneumoniae* BSI in the NICUs of developing countries.

## Disclosure of interest

None declared

